# Subcutaneous Immunization of *Leishmania HSP70-II* Null Mutant Line Reduces the Severity of the Experimental Visceral Leishmaniasis in BALB/c Mice

**DOI:** 10.3390/vaccines8010141

**Published:** 2020-03-23

**Authors:** José Carlos Solana, Laura Ramírez, Emma C. L. Cook, Elena Hernández-García, Silvia Sacristán, M. Elena Martín, Víctor Manuel González, Rosa María Reguera, Rafael Balaña-Fouce, Manuel Fresno, José María Requena, Salvador Iborra, Manuel Soto

**Affiliations:** 1 Centro de Biología Molecular Severo Ochoa (CSIC-UAM), Departamento de Biología Molecular, Nicolás Cabrera 1, Universidad Autónoma de Madrid, 28049 Madrid, Spain; 2 WHO Collaborating Centre for Leishmaniasis, National Centre for Microbiology, Instituto de Salud Carlos III, 28220 Madrid, Spain; 3 Department of Immunology, Ophthalmology and ENT. Complutense University School of Medicine and 12 de Octubre Health Research Institute (imas12), 28040 Madrid, Spain; 4 Departamento de Bioquímica-Investigación, Hospital Ramón y Cajal (IRYCIS), 28034 Madrid, Spain; 5 Departamento de Ciencias Biomédicas, Universidad de León, Campus de Vegazana s/n, 24071 León, Spain

**Keywords:** visceral leishmaniasis, mice, attenuated parasites, vaccine, IFN-γ, T-cells

## Abstract

*Leishmania infantum* parasites cause a severe form of visceral leishmaniasis in human and viscerocutaneous leishmaniasis in dogs. Recently, we reported that immunization with an attenuated *L. infantum* cell line, lacking the *hsp70-II* gene, protects against the development of murine cutaneous leishmaniasis. In this work, we analyzed the vaccine potential of this cell line towards the long-term protection against murine visceral leishmaniasis. This model shows an organ-dependent evolution of the disease. The infection can resolve in the liver but chronically affect spleen and bone marrow. Twelve weeks after subcutaneous administration of attenuated *L. infantum*, Bagg Albino (BALB/c) mice were challenged with infective *L. infantum* parasites expressing the luciferase-encoding gene. Combining in vivo bioimaging techniques with limiting dilution experiments, we report that, in the initial phase of the disease, vaccinated animals presented lower parasite loads than unvaccinated animals. A reduction of the severity of liver damage was also detected. Protection was associated with the induction of rapid parasite-specific IFN-γ production by CD4^+^ and CD8^+^ T cells. However, the vaccine was unable to control the chronic phase of the disease, since we did not find differences in the parasite burdens nor in the immune response at that time point.

## 1. Introduction

Visceral leishmaniasis (VL), the more severe form of leishmaniasis, is caused by parasites of the species *Leishmania donovani* and *Leishmania infantum*. VL constitutes a serious public health problem, causing high morbidity and mortality in different regions of the world. Recent estimates indicate that around 0.4 million new VL cases occur each year. Infections in the Indian subcontinent and in the region of East Africa are caused by *L. donovani*, a species with an anthroponotic transmission cycle [1]. In some *L. donovani* patients, after apparent successful treatment, the infection evolves to a cutaneous clinical outcome, termed post-kala-azar dermal leishmaniasis (PKDL), which favors parasite dissemination by hematophagous sandflies [2]. In contrast, *L. infantum* is the etiological agent causing zoonotic VL in South America and in countries around the Mediterranean [3]. Infected dogs are the principal reservoir for parasite transmission to humans [4]. In dogs, this species also causes a severe disease, named viscerocutaneous canine leishmaniasis (CanL), which shows a wide range of clinical manifestations [5,6]. 

VL is usually fatal if left untreated, and the antileishmanial chemotherapy has serious drawbacks, such as low efficacy and significant toxicity. Although some protein-based vaccines are commercially available in different parts of the world for CanL [7], to date, there is no acceptable vaccine for human VL [8]. Vaccines based on whole leishmanial extracts or recombinant proteins generate a short-term immunity that requires periodic administration of the immunogen in order to maintain circulating effector T cells [9,10]. However, it is well known that both asymptomatic infected individuals and cured patients acquire lifelong immunity to reinfection. For instance, patients who have recovered from cutaneous leishmaniasis (CL) after intradermal inoculation with *L. major* or *L. tropica* are protected against further natural challenge with the same species [11]. Concomitant immunity due to parasite persistence has been correlated with the effector T response maintenance and seems to be responsible for acquired resistance to reinfection [12,13]. These findings, experimentally proved in animal models, would explain the success of leishmanization (inoculation of live parasites), the sole effective vaccination strategy against human leishmaniasis reported to date [11,14]. In an attempt to improve the biosecurity of the live vaccines against CL, as well as to advance the design of vaccines against human VL, some genetically modified attenuated parasite lines have been generated and tested in experimental models of CL and VL [15]. 

Previously, we generated an *L. infantum* line (named *LiΔHSP70-II*) in which both alleles of the *hsp70-II* gene were removed [16]. In its absence, parasites are able to infect, but they have a limited capacity to replicate inside the parasitophorous vacuoles of phagocytic mononuclear cells [17]. More recently, we reported that inoculation of *LiΔHSP70-II* promastigotes caused a local persistent infection that induces short-term and long-term heterologous protection against CL development due to an *L. major* infective challenge in BALB/c and C57BL/6 mice [18,19,20]. This protection correlates with rapid migration of effector T cells producing IFN-γ to the site of the intradermal experimental infection [18]. 

In this article, we tested the ability of the *LiΔHSP70-II* attenuated line in promoting long-term immunity in the BALB/c model of VL. To monitor the development of the infection in different organs [21], we employed a virulent *L. infantum* line genetically expressing a luciferase gene (line *PpyRE9h^+^*), which allows tracking of the parasite distribution by bioluminescent imaging (BLI) techniques [22]. Additionally, at defined time points, we determined the parasite loads using classic titration techniques to monitor disease progression. In parallel, we analyzed the immune responses along the infectious process in control and vaccinated mice.

## 2. Materials and Methods

### 2.1. Mice and Parasites

Female BALB/cOlaHsd mice were purchased from Envigo (Alconbury, Huntingdon, UK) and were 6 weeks old at the beginning of the assays. Animal research complies with EU Directive 2010/63EU, recommendation 2007/526/European Communities (EC) and the Spanish Real Decreto (RD) 53/2013, regarding the protection of animals used for experimental and other scientific purposes. All the procedures were approved by the Government of the Autonomous Community of Madrid (Spain) under the references PROEX 121/14 and PROEX134/19. Procedures were revised by the Animal Care and Use Committee at the Severo Ochoa Molecular Biology Center (reference CEEA-CBMSO 23/243) as well as by the Bioethical Committee of the Spanish Consejo Superior de Investigaciones Científicas under reference 795/2019.

For vaccination, the attenuated *LiΔHSP70-II* line *L*. *infantum* MCAN/ES/96/BCN150 [*Δhsp*70-*II*::*NEO/Δhsp70-II*::*HYG*] was employed [16,18]. Mice were subcutaneously (s.c.) vaccinated with 10^7^ *LiΔHSP70-II* stationary promastigotes (suspended in 30 μL of phosphate saline buffer (PBS)) into the right footpad. Control mice received s.c. the same volume of PBS. For the challenge, mice were intravenously (i.v.) infected with a high dose (10^8^ stationary phase promastigotes in 100 µL of PBS) of *L. infantum* (MCAN/ES/96/BCN150) genetically modified to express the *luc* gene (line *PpyRE9h^+^* [22]). For soluble leishmanial antigen (SLA), freeze-thaw preparations from promastigotes of the wild-type *L. infantum* (MCAN/ES/96/BCN 150) were employed.

Promastigotes of the three strains were cultured at 26 °C in M3 complete medium (M3 medium supplemented with 10% fetal calf serum (FCS; Sigma. St. Louis, MO. USA), 100 U/mL of penicillin, and 100 μg/mL of streptomycin). For in vitro growth of the *LiΔHSP70-II* or the *PpyRE9h^+^* lines, M3 complete medium was supplemented with 20 μg/mL of G418 and 50 μg/mL of hygromycin or with 100 μg/mL of puromycin, respectively. All antibiotics were purchased from Thermo Fischer Scientific (Waltham, MA, USA).

### 2.2. Follow-up of in Vivo Infections by BLI

For BLI, animals infected with the *PpyREh9^+^ L. infantum* line were monitored weekly in a Charge-Coupled Device (CCD) IVIS 100 Xenogen system (Caliper Life Science, Hopkinton, MA, USA) [22]. Mice were intraperitoneally injected with D-luciferin (150 mg/Kg; Perkin Elmer (Waltham, MA, USA)) and anesthetized with isoflurane before acquiring the images for a period of 10 min. To estimate the parasite burden in living mice, the radiance values of the images corresponding to the liver (ventral view) were considered regions of interest (ROIs). Regions of interest (ROIs) were drawn using Living Image v.4.3 to quantify BLI expressed as radiance (p/s/cm^2^/sr).

### 2.3. Quantification of Parasites in Liver, Spleen, and Bone Marrow (BM) by Limiting Dilution

Parasite loads were determined by limiting dilution as described elsewhere [23]. The spleen, the BM perfused from the femur cavity, or a piece of approximately 20 mg of liver were obtained from each mouse and individually processed by filtration through 70-μm cell strainers (Corning Gmbh, Kaiserslautern, Germany). Cells were cultured at 26 °C in M3 complete medium prepared as described above but supplemented with 20% FCS. To determine parasite numbers belonging to the *PpyREh9^+^ L. infantum* line, puromycin (100 μg/mL) was added to the medium. Moreover, to determine the parasite load due to the attenuated *LiΔHSP70-II* line, medium was supplemented with hygromycin (50 μg/mL) and geneticin (G418; 20 μg/mL). The presence of the attenuated line was also monitored in the lymph node draining the site of infection (right popliteal lymph node). Serial dilutions of the cells (1/3) were performed in 96-well flat-bottomed microtiter plates (Thermo Fischer Scientific). The number of viable parasites was determined from the highest dilution at which promastigotes could be observed after 10 days of incubation at 26 ℃. Values are represented per g of liver, per 1 × 10^7^ BM cells, or per the whole spleen or lymph node.

### 2.4. Serum Preparation and Analysis of the Humoral Responses

Serum was obtained from blood samples taken at week 1 (silent phase), week 5 (initial phase), and week 11 (late phase) after the infective challenge. The reactivity against SLA was determined by ELISA as described in [18]. Serum samples were serially diluted two-fold starting with from 1/50. All samples were analyzed individually. Anti-IgG1 or anti-IgG2a horseradish peroxidase-conjugated anti-mouse immunoglobulins from Nordic (BioSite, Täby, Sweden) were employed as secondary antibodies (1/2000 dilution). Orto-phenylenediamine was used for color development. Optical density values were read at 490 nm in an ELISA microplate spectrophotometer (Model 680, Bio-Rad Laboratories, Hercules, CA, USA). The reciprocal end-point titer was defined as the inverse value of the highest serum dilution factor giving an absorbance value higher than >0.1.

### 2.5. Determination of Cytokine Concentrations in Culture Supernatants

Primary cultures from the spleen of mice were established in Roswell Park Memorial Institute (RPMI) complete medium: RPMI medium (Sigma, Sigma. St. Louis, MO. USA) supplemented with 10% heat-inactivated FCS, 20 mM L-glutamine, 200 U/mL penicillin, 100 μg/mL streptomycin, and 50 μg/mL gentamicin (Thermo Fischer Scientific). Spleen cells (2 × 10^6^ cells/mL) were cultured without stimulus, or incubated with SLA (12 μg/mL) at 37 °C and 5% CO_2_ for 72 h. The levels of IFN-γ, Interleukin-10 (IL-10), and IL-4 in culture supernatants were determined by sandwich ELISA following the manufacturer’s instructions (Thermo Fisher Scientific). 

### 2.6. Cell Cytometry Analyses

Granulocyte Macrophage Colony-Stimulating Factor (GMCSF) BM-derived cells (GM-DCs cells) were obtained from BM suspensions obtained from naïve mice by culturing for 7 days in RPMI complete medium supplemented with 20 ng/mL recombinant GMCSF (Peprotech, London, UK). GM-DCs cells were pulsed with *L. infantum* SLA (1 µg/mL) for the last 24 h of culture to obtain stimulated cells. For the analysis of percentages of IFN-γ-producing spleen cells, splenocyte cultures were established in RPMI complete medium as described above. Spleen cells (2 × 10^6^ cells/mL) were co-cultured at 37 °C and 5% CO_2_ with GM-DCs cells stimulated or not with SLA (4 × 10^5^ cell/mL) for 24 h. Co-cultures were treated with 10 µg/mL brefeldin A (Sigma) for the last 6 h of culture. After, cells were collected, washed in PBS supplemented with 1% heat-inactivated FCS (PBSw), and incubated with Mouse Fc Block (BD Biosciences, San José, CA, USA) prior to staining. For the staining of the surface markers, cells were incubated with antibodies specific for CD3 (clone 145-2C11; APC), CD4 (clone RM4-5; BV570), and CD8 (clone 53-6.7; FITC) during 30 min at 4 °C. After washing in PBSw, cells were fixed and permeabilized with Cytofix/Cytoperm (BD Biosciences). Next, PE/Cy7 anti-mouse IFN-γ (clone XMG1.2) antibody was added for 30 min at 4 °C. Finally, cells were washed and analyzed. Antibodies were purchased from BioLegend (San Diego, CA, USA). Samples were analyzed using a FACS Canto II flow cytometer and FACSDiva Software (BD Biosciences, San José, CA, USA) and processed and plotted with FlowJo Software (FlowJo LLC, Ashland, Oregon, OR, USA). In all the assays, samples were collected and processed individually.

### 2.7. Statistical Analysis

Statistical analysis was performed using the Graph-Pad Prism 5 program. The Shapiro–Wilk normality test was employed when samples were n ≥ 7. Parametric data were analyzed by a two-tailed Student t-test. Non-parametric data (or data with n < 7) were analyzed by a Mann–Whitney test. Differences were considered significant when *p* < 0.05.

## 3. Results

### 3.1. Comparative Analysis of Evolution of Leishmania Infection in Vaccinated and Control Mice

**Figure 1:**
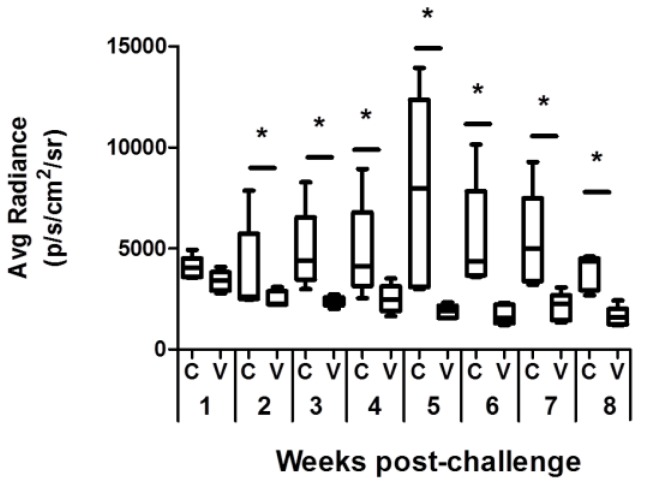
Evaluation of the *L. infantum* infection by in vivo bioluminescent imaging (BLI). BALB/c mice (*n* = 5 per group) were inoculated s.c. (left footpad) with PBS (control group) or vaccinated with 10^7^ *LiΔHSP70-II* attenuated parasites prepared in PBS. Twelve weeks after inoculation, animals were challenged i.v. with 10^8^ *PpyRE9h^+^ L. infantum* infective promastigotes. Radiance data are represented as box and whisker (min to max) plots. Statistics were obtained using a Mann–Whitney test. * shows the statistical differences between control and vaccinated mice (*p* < 0.05). Results are representative of two independent experiments.

**Figure 2:**
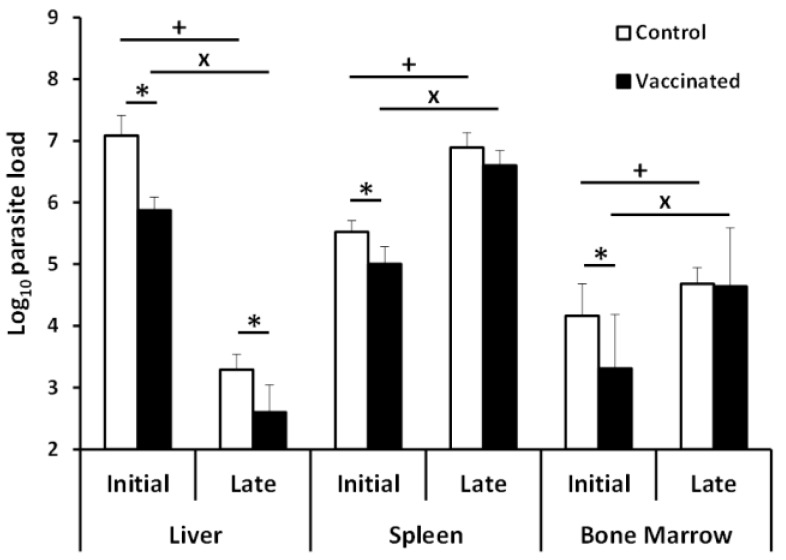
Parasite load monitoring in the liver, spleen, and bone marrow. The presence of viable parasites of the *PpyRE9h^+^ L. infantum* line in control and vaccinated mice was analyzed by limiting dilution in the presence of puromycin at week 5 (initial phase; *n* = 8) and at week 11 (late phase; *n* = 8) after challenge with 10^8^ promastigotes. Samples from each mouse were processed and analyzed independently. The graph shows the mean (+SD) of parasites per g in the liver, spleen (total organ), or parasites per 1 × 10^7^ bone marrow cells. * (*p* < 0.05) shows the statistical differences between control and vaccinated mice. **^+^** (*p* < 0.05) shows the statistical differences between the initial and late phases of control mice. **^×^** (*p* < 0.05) shows the statistical differences between the initial and late phases of vaccinated mice. All samples were analyzed using a Student T test. Results are representative of at least two independent experiments.

To analyze the effect of vaccination with the *LiΔHSP70-II* attenuated line in the evolution of experimental VL, we challenged control and vaccinated mice groups with 10^8^ stationary promastigotes of an *L. infantum* virulent strain. The challenge was done 12 weeks post-vaccination in order to analyze long-term protection. To do a weekly follow-up of the infection avoiding the weekly killing of mice, we used a virulent *L. infantum* line expressing the *luc* gene (*PpyRE9h^+^* line). The radiance values of infected mice were monitored by BLI, taking pictures of the animals in the supine position. From week 2 to week 8 (end of the assay), the radiance values in the vaccinated mice were significantly lower than the values emitted in the control group (Figure 1 and Supplementary Figure S1). These data indicated that vaccination would be restricting parasite multiplication, at least in the liver, which is the organ more clearly depicted by the luminescence emission (see Supplementary Figure S1). To assess the parasite loads in the main targets of viscerotropic *Leishmania* species (i.e., liver, spleen, and bone marrow (BM)), in vitro limiting dilution assays in the presence of puromycin (to select only *PpyRE9h+* parasites) were carried out in animals sacrificed at 5 weeks (initial phase of infection) and 11 weeks (chronic late phase [21]) post-challenge [24]. Vaccinated mice showed significant reductions in the parasite loads regarding control mice in the liver, spleen, and BM at the initial phase (Figure 2, initial). At the late phase, the number of parasites remained significantly lower only in the liver of the vaccinated mice, whereas similar numbers of parasites were found in the spleen and BM in both mice groups (Figure 2, late). A decrease in the number of parasites in the liver was detected throughout the course of the disease (Figure 2, liver). On the contrary, parasite numbers incremented from the initial period to the late phase in the spleen and in the BM of both mice groups (Figure 2; spleen and BM).

In parallel, limiting dilution assays were performed in which tissue samples were cultured in medium containing hygromycin and geneticin to select the LiΔHSP70-II parasites used for vaccination. Remarkably, parasites of the attenuated line were only found in the draining lymph node (popliteous) of the site of vaccination (right footpad) while no parasites were detected in the internal organs from vaccinated mice and, as expected, in animals from the control group (Supplementary Figure S2).

The analysis of the histopathological lesions at the late phase of disease (11 weeks post-challenge) revealed that the control mice presented a greater number of granulomatous formations in the liver than the vaccinated animals (Figure S3). These data are consistent with the lower parasitic load detected in the vaccinated animals along the period of infection (Figure 2; liver). In summary, the hepatic parasite loads and histological images suggest that vaccination with the attenuated line reduced the severity of the infection. 

### 3.2. Vaccinated Animals Showed an Earlier Humoral Response against Leishmanial Antigens after Infective Challenge

In order to study the immune response elicited by the vaccination with the attenuated line, we first analyzed the humoral response at three time points post-infection: At the beginning of infection (1 week after challenge), when parasite replication does not induce detectable parasite-specific pro-inflammatory responses in the spleen (silent phase; one week post-challenge); at the initial phase, when spleen cells recover their capacity to produce IFN-γ and the effector phase in the liver begins, characterized by the formation of granulomas and the decrease in hepatic parasitic load (initial phase; 5 weeks post-challenge); and at the chronic phase (11 weeks post-challenge), when parasites are eliminated in the liver but their multiplication continues in the spleen and BM [21].

**Figure 3:**
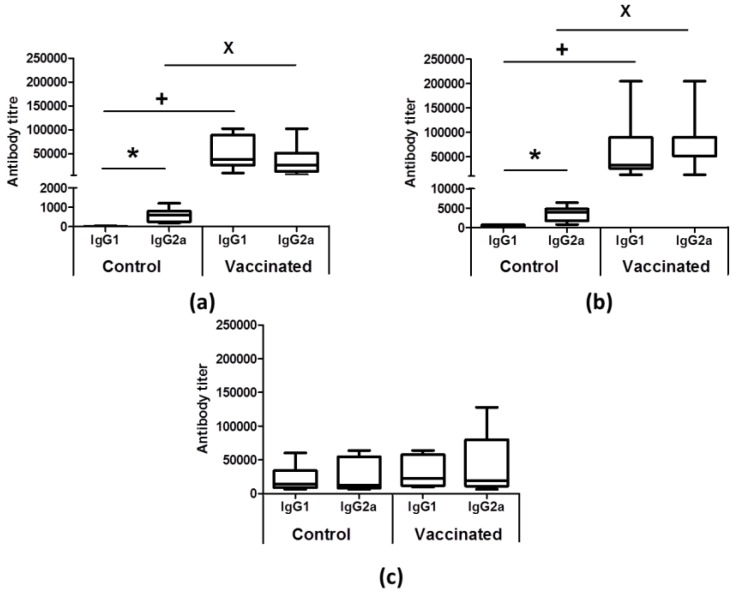
Vaccinated animals develop an earlier parasite-specific antibody response than controls after infective challenge. The reciprocal end-point titer of IgG1 or IgG2a against leishmanial antigens was determined by ELISA from sera samples taken at the silent phase (**a**; 1 week post-challenge), at the initial phase (**b**; 5 weeks post-challenge), and at the late phase of infection (**c**; 11 weeks post-challenge). Sera samples from control (n = 8) and vaccinated (*n* = 8) animals were studied individually. Data are represented as box and whisker (min to max) plots and statistics were analyzed by a Mann–Whitney test. * (*p* < 0.05) shows the statistical differences between IgG1 and IgG2a titers in the control group. **^+^** (*p* < 0.05) or ^×^ (*p* < 0.05) shows the statistical differences between vaccinated and control groups of IgG1 or IgG2a titers, respectively. Results are representative of two independent experiments.

We first analyzed the anti-*Leishmania* humoral response, determining the titer of IgG1 and IgG2a anti-SLA antibodies in the sera of infected mice. Mice from the control group showed very low titers of antibodies against parasite proteins at both the silent and the initial phase, the IgG2a antibodies being the predominant subclass (Figure 3a,b). Later, in animals from the control group, the title of both subclasses increased, and in the chronic phase, we did not detect statistically significant differences between both titers (Figure 3c). In vaccinated animals, IgG1 and IgG2a anti-SLA titers were significantly higher than those observed in the control group at the silent and at the initial phases (Figure 3a,b). Comparison of the anti-SLA antibody response before (Figure S4) and after the challenge with the *L. infantum PpyRE9h^+^* line (Figure 3a,b) demonstrated that infection boosted the production of both IgG1 and IgG2a subclasses in the vaccinated animals at the early stages of infection. At the chronic phase, the humoral response against the parasite in the vaccinated group was similar to that of the control group (Figure 3c).

### 3.3. Vaccination with the Attenuated LiΔHSP70-II Line Anticipates the Parasite-Specific Cellular Immune Response after the Infective Challenge

**Figure 4:**
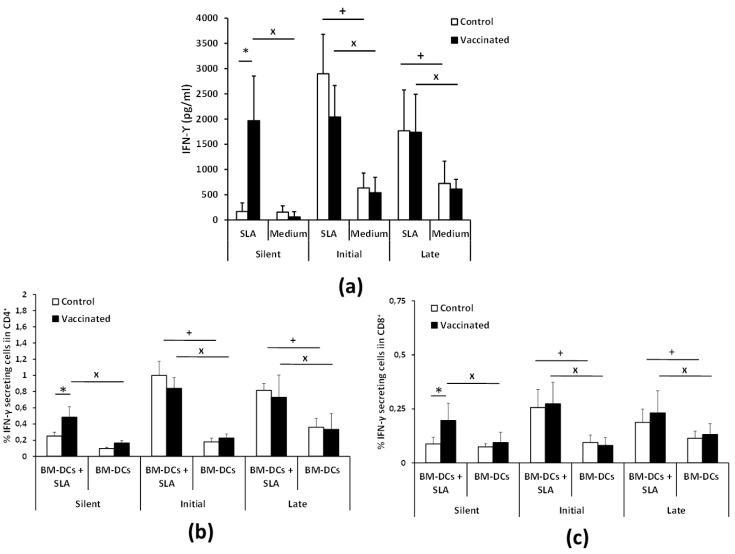
Analyses of parasite-specific IFN-γ production. BALB/c mice (n = 8 per group) were inoculated s.c. in the left footpad with PBS (control group) or vaccinated with 10^7^ *LiΔHSP70-II* attenuated parasites prepared in PBS. Twelve weeks after inoculation, animals were challenged i.v. with 10^8^ *PpyRE9h^+^ L. infantum* infective promastigotes. Spleen cell cultures from each mouse were independently established at the silent phase (1 week post-challenge), at the initial phase (5 weeks post-challenge), and at the late phase of infection (11 weeks post-challenge) and stimulated (SLA) or not (medium) with soluble leishmanial antigens for 72 h. IFN-γ levels were measured in culture supernatants by quantitative sandwich ELISA (**a**). Spleen cell cultures established at the indicated phases were independently stimulated for 24 h with BM-DCs pulsed or not with SLA. Afterwards, cells were processed for flow cytometry. The percentages of IFN-γ-secreting cells in CD4^+^ (**b**) or CD8^+^ (**c**) gates are shown. All data are represented as the mean (+ SD) and statistics were analyzed by a Student T test. * (*p* < 0.05) shows the statistical differences between control and vaccinated mice. ^+^ (*p* < 0.05) shows the statistical differences between SLA stimulation and non-stimulation in the control group. ^×^ (*p* < 0.05) shows the statistical differences between SLA stimulation and non-stimulation in the vaccinated group. Results are representative of two independent experiments.

To investigate the cellular immune response elicited against the parasite, we analyzed the level of *Leishmania*-specific cytokines secreted by spleen cells in vitro taken at the three phases after the infective challenge described above. At the silent phase, SLA-specific production of IFN-γ in culture supernatants was only observed in vaccinated mice. Later on, at the initial and late phases of the disease, splenocytes from both animal groups secreted similar levels of this cytokine (Figure 4a). We found that antigen-stimulated CD4^+^ and CD8^+^ T cells produced IFN-γ at the silent phase in vaccinated mice but not in the control group (Figure 4b,c). At later stages of infection, both T cell types were implicated in the SLA-specific secretion of IFN-γ in control and vaccinated mice (Figure 4b,c and Supplementary Figure S5). We conclude that vaccinated animals have an earlier IFN-γ-mediated response to the parasite in which both CD4^+^ and CD8^+^ T lymphocytes are involved.

**Figure 5:**
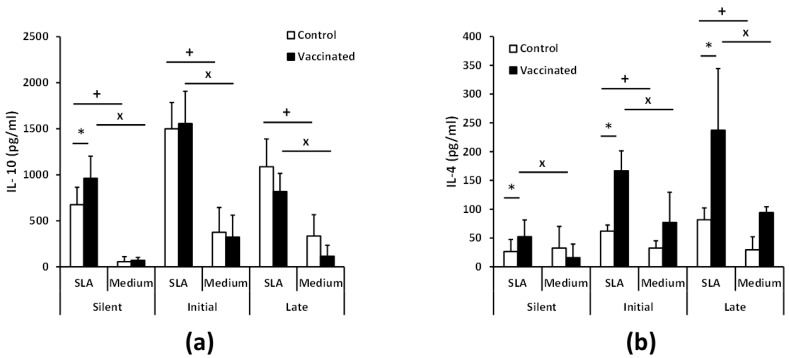
Parasite-specific IL-10 and IL-4 production. BALB/c mice (*n* = 8 per group) were vaccinated and infected as in Figure 4. Spleen cell cultures from each mouse were independently established in the silent, initial, and late phases and stimulated for 72 h with soluble SLA or cultured without stimulus (medium). IL-10 (**a**) or IL-4 (**b**) levels were measured in culture supernatants by quantitative sandwich ELISA. Data are represented as the mean (+ SD) and statistics were analyzed by a Student T test. * (*p* < 0.05) shows the statistical differences between control and vaccinated mice. ^+^ (*p* < 0.05) shows the statistical differences between SLA-stimulated and unstimulated cytokine production in the control group. ^×^ (*p* < 0.05) shows the statistical differences between SLA-stimulated and unstimulated levels in the vaccinated group. Results are representative of two independent experiments.

We detected the presence of parasite-specific IL-10 in the culture supernatants of spleen cell cultures derived from control and vaccinated animals from the silent to the chronic phase. The early production of this cytokine was significantly higher in the vaccinated group at the silent phase, although similar levels of IL-10 were produced in both groups as the disease progressed to the initial and chronic phases (Figure 5a). 

On the other hand, the post-challenge production of IL-4 occurred earlier and with increased levels in the vaccinated animals than in the control ones. Thus, in the silent phase, we detected parasite-specific IL-4 only in cultures established from the vaccinated mice. In addition, the levels of IL-4 were significantly increased in vaccinated animals compared to control ones at the initial and chronic phases (Figure 5b). 

In conclusion, vaccination with *LiΔHSP70-II* attenuated parasites decreased the load of *PpyRE9h+ L. infantum* parasites in our mouse model. It also increased humoral responses and IFN-γ production by CD4^+^ and CD8^+^ T cells in the silent phase of the disease while inducing IL-4 production in the spleen throughout the duration of the disease.

## 4. Discussion

During the last 20 years, different approaches have been pursued to obtain anti-*Leishmania* prophylactic vaccines. The identification of defined leishmanial antigens or parasite extracts with antigenic proteins has allowed the formulation of some commercially available veterinary vaccines [25]. In addition, some of these vaccines are under study in clinical trials to prevent human leishmaniasis [26,27]. The most important limitation of subunit vaccines is the short-term protection induced. To maintain immunity, these vaccines require booster doses, since a transient effector T cell response against the proteins composing the vaccine precludes the induction of long-term immunity [28,29]. On the contrary, the balanced effector/memory T response induced by the infection with virulent parasites can be maintained by parasite persistence, resulting in long-term immunity as it happens in patients vaccinated by the inoculation of *L. major* virulent parasites (leishmanization) [11,28,30].

Vaccination with genetically attenuated parasite cell lines has the objective of being as effective as leishmanization [31], avoiding the problems derived from using a non-attenuated parasite [32]. In this work, we analyzed the prophylactic properties against VL of a live vaccine based on an *L. infantum* genetically attenuated cell line *LiΔHSP70-II* [17]. The main reason for analyzing the immune-prophylactic properties of this attenuated cell line in a VL model was related to the long-term robust protection generated by its inoculation against CL due to experimental infection with *L. major*. Protection was observed in a resistant model that mimics human CL, namely C57BL/6 mice challenged in the ear dermis with a low dose of metacyclic promastigotes [33]. The *LiΔHSP70-II*-based vaccine also induced protection in the progressive form of leishmaniasis developed in the susceptible BALB/c mice strain when infected with the *L. major* model [18,20], a model that may reflect in part some aspects of the human VL disease [34]. It is clear that the experimental challenge of mice with viscerotropic species does not fully reproduce the peculiarities of human VL patients because of the different organ-dependent evolution of the disease [35]. Whereas the granulomatous response in the liver seems to follow many of the characteristics observed in asymptomatic patients, the spleen can be considered as a model of the pathology and immune dysfunction in the progressive disease found in human VL and CanL patients [36].

To have a more complete view of the protective capabilities of this live vaccine, we tested the effects of the *LiΔHSP70-II* administration in a murine (BALB/c) model of infection with *L. infantum*, since murine models of VL have been considered suitable for the evaluation of anti-leishmanial vaccine candidates [37]. To make a continuous follow-up of the disease, determining the time of the initial (coinciding with a peak of the hepatic parasite burdens in the liver) or the late phases (hepatic immunity and chronic infection of the spleen and BM), we employed as a challenge infective promastigotes of the *L. infantum PpyRE9h^+^* cell line. In these parasites, the synthesis of the luciferase enzymatic activity allows the in vivo detection of the presence of *Leishmania* in the internal organs and the determination of parasite burdens employing BLI techniques [22]. This approach allowed us to monitor disease evolution, reducing the number of animals used in the assays [38]. However, this approach has supposed the limitation of having to use a high infective dose (1 × 10^8^ stationary phase promastigotes) to correctly detect parasites. In these experimental conditions, our results show that the vaccinated animals had a less severe development of the disease than control animals, especially in the hepatic manifestations. A comparison between vaccinated and control mice revealed statistically significant differences in the parasitic loads found in the liver (BLI values) (Figure 1). These observations were validated by the determination of viable parasites by limiting dilution at the initial and the late phases of the disease (Figure 2). In addition, the low number of granulomas found in the liver of the vaccinated mice at the end of the effector phase points to a limited hepatic infection, since the resolution of the parasitic load in the liver largely depends on the formation of these inflammatory active structures assembled around parasitized Kupffer cells [39]. The production of parasite-dependent IFN-γ responses in the spleen is essential for the generation of hepatic granulomas [40,41] and the destruction of parasites by infected macrophages [42,43]. Both CD4^+^ and CD8^+^ T cells have been implicated in the production of this cytokine in murine experimental VL models [44,45]; the protection generated by different vaccine strategies having correlated with the production of IFN-γ [46]. Our observations showed that after infection, vaccinated animals mounted an early parasite-specific IFN-γ-mediated response (Figure 4). In the silent phase of infection, control group mice produced significantly less parasite-specific IFN-γ. However, at that time point, we were able to detect CD4^+^ T cells secreting IFN-γ upon stimulation with SLAs in the spleen of the vaccinated animals (Figure 4b). The early involvement of this cell type in the generation of protection against infection with *L. infantum* has also been demonstrated in mice (C57BL/6) leishmanized with *L. major* [47]. In our experiments, the involvement of CD8^+^ T lymphocytes in IFN-γ secretion was also appreciated, although in a smaller percentage than CD4^+^ T cells (Figure 4b,c). The rapid response elicited in the vaccinated animals suggests the implication of concomitant immunity. Based on data from our prior investigation, the vaccine was administrated subcutaneously (right footpad) to induce a persistent infection in the draining popliteous lymph node without affecting the internal organs [18]. The presence of persistent parasites in the lymph node was confirmed in this work, since we were able to detect *LiΔHSP70-II* parasites at the initial and at the late phases after infective challenge. Remarkably, parasites composing the vaccine were confined to this location and we could not detect their presence in the other analyzed organs: Liver, spleen, or BM (Figure S2). This observation highlights the attenuated nature of the *LiΔHSP70-II* line as described for other genetically attenuated viscerotropic *Leishmania* lines: *LdCen^-/-^*, a *L. donovani* line deficient in a calcium-binding protein related with the cytoskeleton termed centrin [48]; or *Ldp27^-/-^,* a cell line that lacks a protein part of the cytochrome c oxidase complex [49] and is derived from the same species. Finally, it should be noted that in the silent phase, the vaccinated animals also showed a greater production of IL-10 and IL-4 than control animals (Figure 5) as well as higher titers of parasite-specific IgG1 and IgG2a antibodies (Figure 3). We conclude that the typical mixed pro- and anti-inflammatory immune response found in BALB/c mice after *L. infantum* challenge [50] that was observed after vaccination with the *LiΔHSP70-II* cell line [18] was boosted after challenge with the *L. infantum PpyRE9h^+^* infective promastigotes.

The protection induced in the initial phase of the disease, evidenced by the lower hepatic involvement mentioned above, and by the presence of a lower number of parasites in the spleen or BM of vaccinated animals with respect to control ones at week 5 post-challenge (Figure 2) is lost in the late (chronic) phase of the disease. Our results show that at the end of the assay, there are no differences between control and vaccinated animals in the number of parasites in the spleen or BM. These data contrast with those published after administration of the *LdCen^-/-^* or *Ldp27^-/-^*-based vaccines, where animals showed a reduction in the splenic parasitic burden in the chronic phase of the disease (10–12 weeks) [49,51]. These differences in the degree of protection could be explained by the evolution of cytokine patterns secreted by animals vaccinated upon stimulation with SLA. In contrast to the silent phase, in the early and late phases, IFN-γ levels secreted by splenocytes stimulated with SLA were similar between vaccinated and control animals (Figure 4). Something similar occurs with the production of IL-10 (Figure 5a), a cytokine related to VL pathology [52,53,54]. These results have as a consequence that the IFN-γ/IL-10 ratio is not elevated in vaccinated animals during the chronic phase of infection. This parameter was correlated with protection after parasite inoculation in a live vaccine based on *L. infantum* parasites deficient in SIR2 (*LiSir2^+/-^*), a protein member of the conserved eukaryotic homologous of Sir two (Hst) proteins [55]. In addition, the level of IL-4 secreted in response to parasitic extracts also remains higher in vaccinated animals than in controls (Figure 5b). The generation of *Leishmania*-specific IL-4 responses does not appear to be associated with susceptibility in murine models of VL [56,57]. On the other hand, the protective immunity against VL showed after vaccination with *L. donovani* parasites deficient in one enzymatic activity implicated in ascorbic acid metabolism was correlated to the control of IL-4 responses [58], and a parasite-dependent production of IL-4 has been associated with the failure of some subunit-based vaccines [59]. Therefore, we cannot discard a negative effect on the production of this cytokine in the chronic phase of infection in vaccinated animals. These results do not necessarily rule out the use of the vaccine based on the *LiΔHSP70-II* line due to the experimental differences existing between these different vaccination trials with the one used in this work. These differences can have an important influence on the development of the VL [21,60], which prevents a direct comparison of the results. Moreover, models employing needle inoculation of the infective agent simplify the infective process and obviate the natural transmission from the sand fly to the skin. Thus, in most of the cases, challenge was performed intravenously (this work, and [49,51,58]) or intraperitoneally [55]. Among these differences, we want to highlight the high infective dose used in this work, 1 × 10^8^ promastigotes administered intravenously. In this regard, and taking as an example, a canine vaccine that has proven its efficacy against natural CanL in a field assay [61] when tested in experimental models was able to protect dogs against an intravenous challenge with 5 × 10^5^ promastigotes [62] but failed to protect animals challenged when the inoculum was increased by two orders of magnitude [63].

## 5. Conclusions

Our results demonstrated that the *LiΔHSP70-II*-based vaccine, in addition to generating robust protection against *L. major* heterologous challenge [18,20], is capable of contributing to controlling the acute phase of the disease, characteristic of the experimental murine model of VL. Thus, vaccinated mice presented limited parasite loads in the liver, associated with a decrease in the spleen and BM parasite burdens in comparison to unvaccinated controls at the initial stage of infection. This homologous protection was associated with a rapid IFN-γ response mediated by CD4^+^ and CD8^+^ effector T cells in the spleen. The rapid response in the liver was similar to that observed in the dermis and the retromandibular lymph nodes after *L. major* intradermal challenge in the ears of vaccinated mice [18]. As it was also described for C57BL/6 mice protected against VL after leishmanization (vaccination with infective *L. major* parasites) [47], we can conclude that the maintenance of effector T cells by concomitant immunity is related to the protective capacity of the vaccine demonstrated at the acute phase of infection. Further assays should be performed using alternative experimental conditions in mice or other animal models, in order to know the real potential of this vaccine strategy in the development of vaccines against different forms of human leishmaniasis.

## Supplementary Materials

The following are available online at https://www.mdpi.com/2076-393X/8/1/141/s1, Figure S1: In vivo evaluation of *L. infantum* infection. Ventral view images of mice from the experiment described in Figure 1 are shown. Pseudocolour heat maps indicate the intensity of bioluminescence from low (blue) to high (red). All images use the same scale heat map (indicated to the right). Images were taken at the indicated weeks post-challenge with 1 × 10^8^ *PpyRE9h+ L. infantum* infective promastigotes, Figure S2. Monitoring of the presence of the *LiΔHSP70-II* attenuated line in mice affected by VL. The presence of viable parasites of the *L. infantum* attenuated line was analyzed in the liver, spleen, bone marrow, and the lymph node draining the site of infection (right popliteal) in control and vaccinated mice after the infective challenge with *PpyRE9h^+^ L. infantum*. Limiting dilution cultures were performed in the presence of hygromycin and geneticin at week 5 (initial phase; *n* = 8) and at week 11 (late phase; *n* = 8). Samples from each mouse were processed and analyzed independently. The graph shows the mean (+SD) of parasites per total popliteal lymph node. Results are representative of at least two independent experiments, Figure S3. Histopathological alterations of the liver at the late phase of infection. Liver sections stained with H&E of a representative member of the control or the vaccinated mice at week 11 post-challenge (4× and 20× magnification), Figure S4. Anti-SLA response before challenge with the infective *PpyRE9h^+^ L. infantum* line. Reciprocal end-point titer of IgG1 or IgG2a against leishmanial antigens was determined by ELISA from sera samples taken 12 weeks after inoculation with saline (control) or the *L. infantum* attenuated line (vaccinated). Sera samples (*n* = 11 per group) were studied individually. Data are represented as box and whisker (min to max) plots. Results are representative of two independent experiments, Figure S5. Analysis of splenic T cell populations. (a) Representative panels and gating strategy of experiments performed for obtaining data from Figure 4. (b) Fluorescence minus one control (FMO controls).
