# Supplementary figures and images for: Subcutaneous Immunization of Leishmania HSP70-II Null Mutant Line Reduces the Severity of the Experimental Visceral Leishmaniasis in BALB/c Mice
Source: Vaccines (Basel). 2020 Mar 23;8(1):141. doi: 10.3390/vaccines8010141 (PMC7157689; doi:10.3390/vaccines8010141)

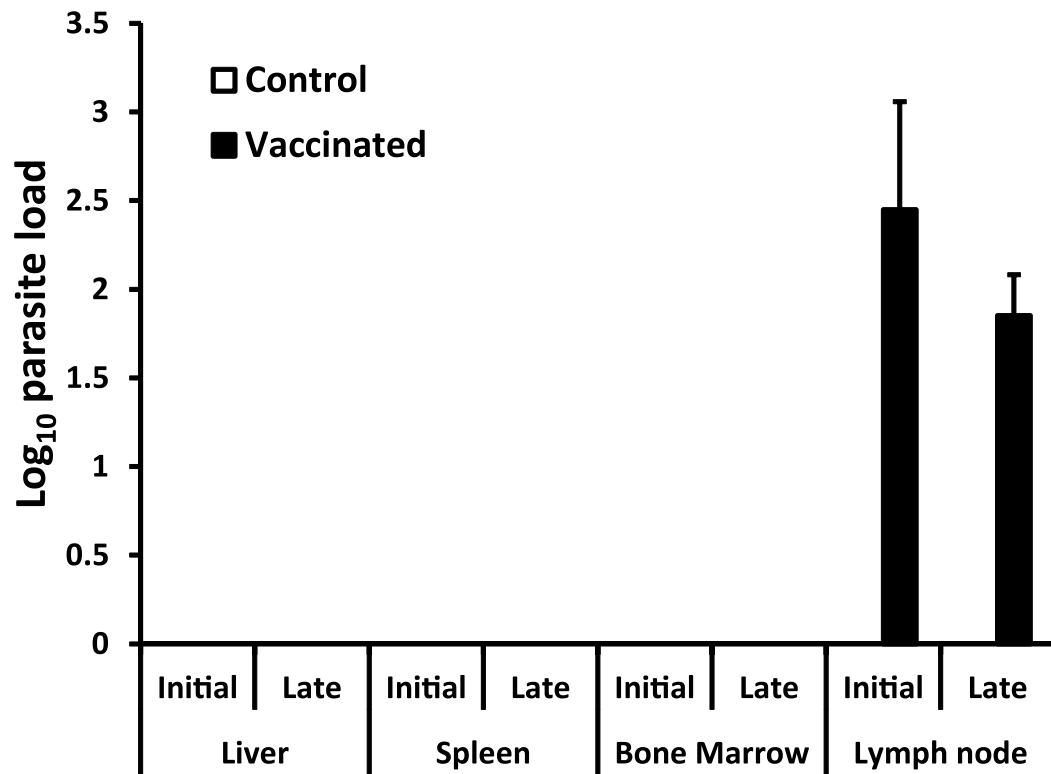

Supplement: Supplementary file 1 [file vaccines-08-00141-s001.zip › Supplementary/SupplFig2.pdf]

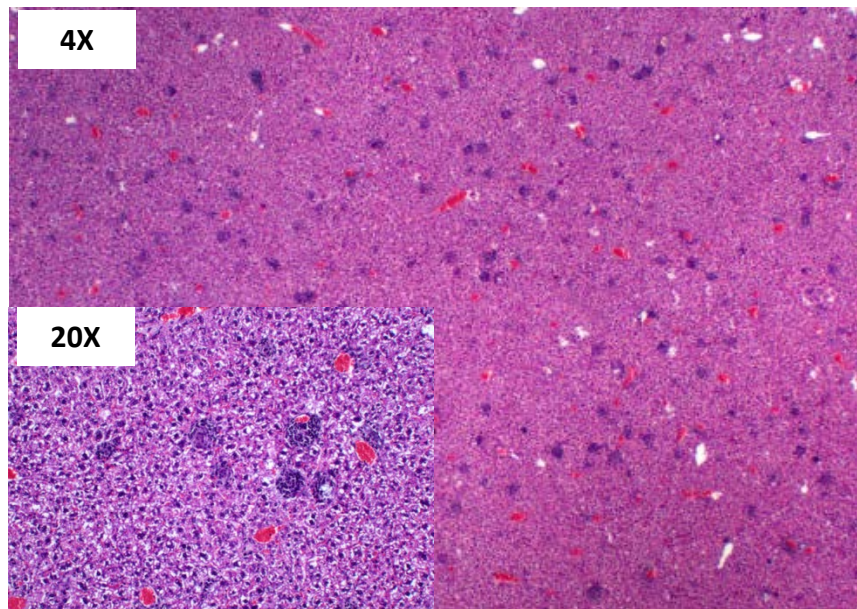

**Control**

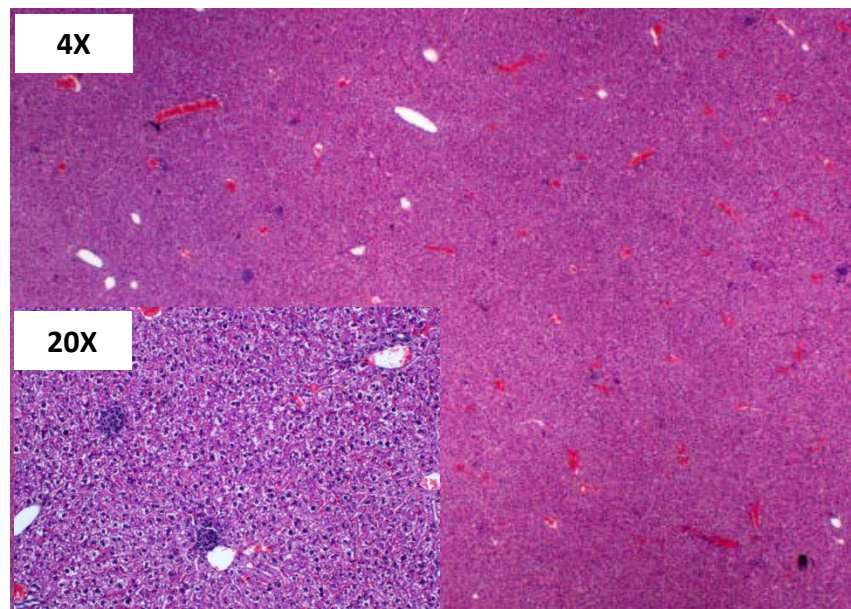

**Vaccinated**

Supplement: Supplementary file 1 [file vaccines-08-00141-s001.zip › Supplementary/SupplFig3.pdf]

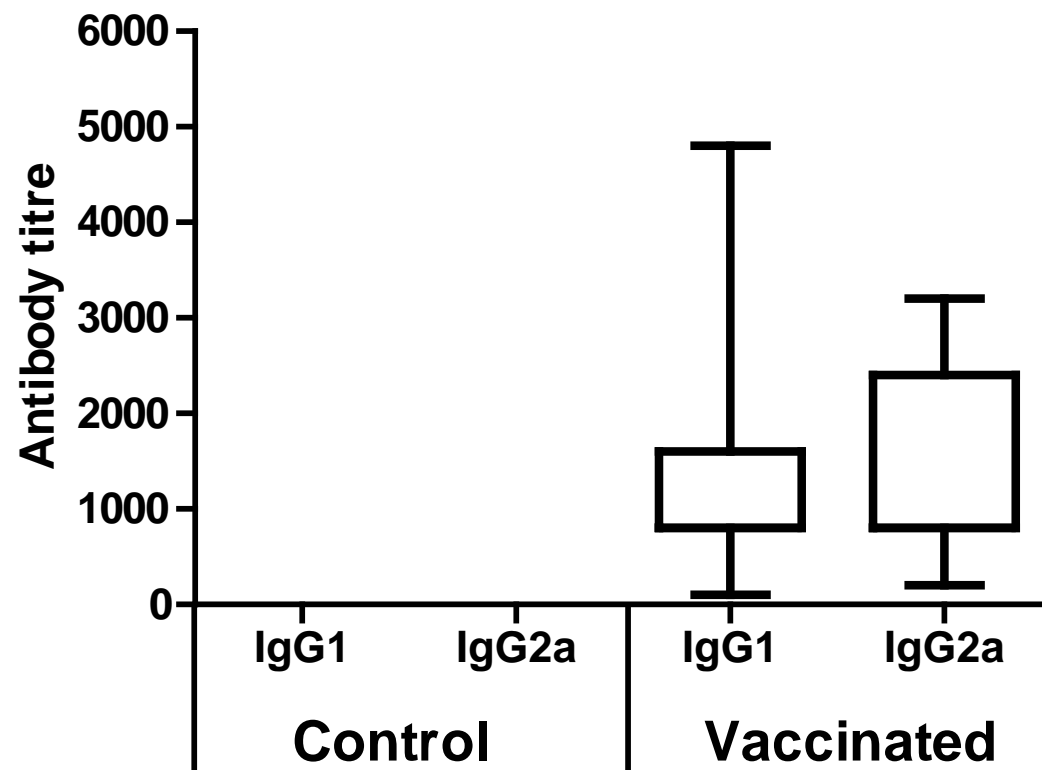

Supplement: Supplementary file 1 [file vaccines-08-00141-s001.zip › Supplementary/SupplFig4.pdf]

(a)

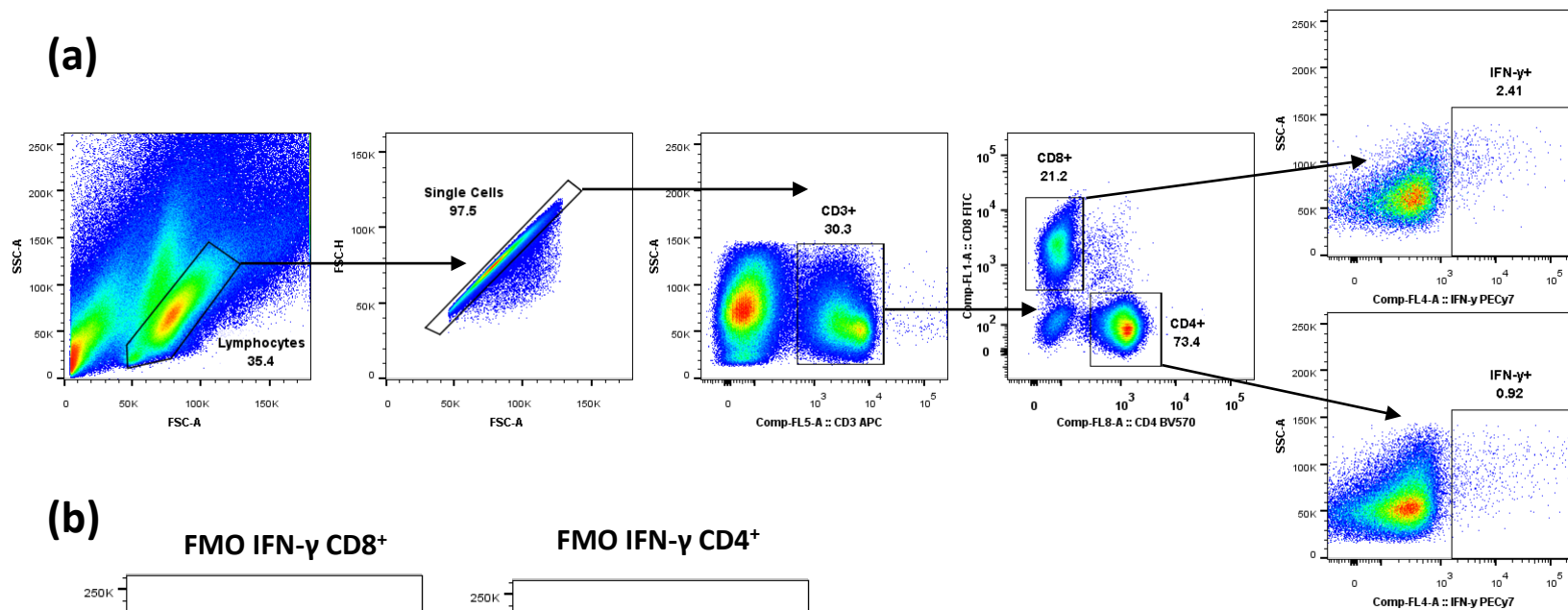

(b)

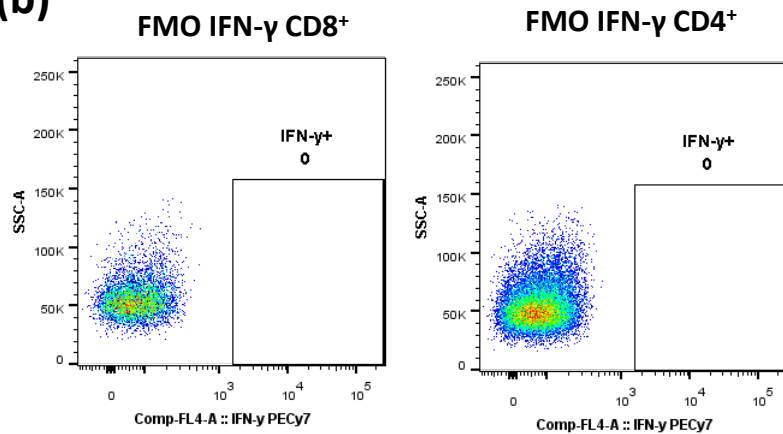

Supplement: Supplementary file 1 [file vaccines-08-00141-s001.zip › Supplementary/SupplFig5.pdf]
