# Supplementary material for: Subcutaneous Immunization of Leishmania HSP70-II Null Mutant Line Reduces the Severity of the Experimental Visceral Leishmaniasis in BALB/c Mice
Source: Vaccines (Basel). 2020 Mar 23;8(1):141. doi: 10.3390/vaccines8010141 (PMC7157689; doi:10.3390/vaccines8010141)
Supplement: Supplementary file 1 [file SupplFig1.pdf › Supplementary/SupplFig1.pdf]

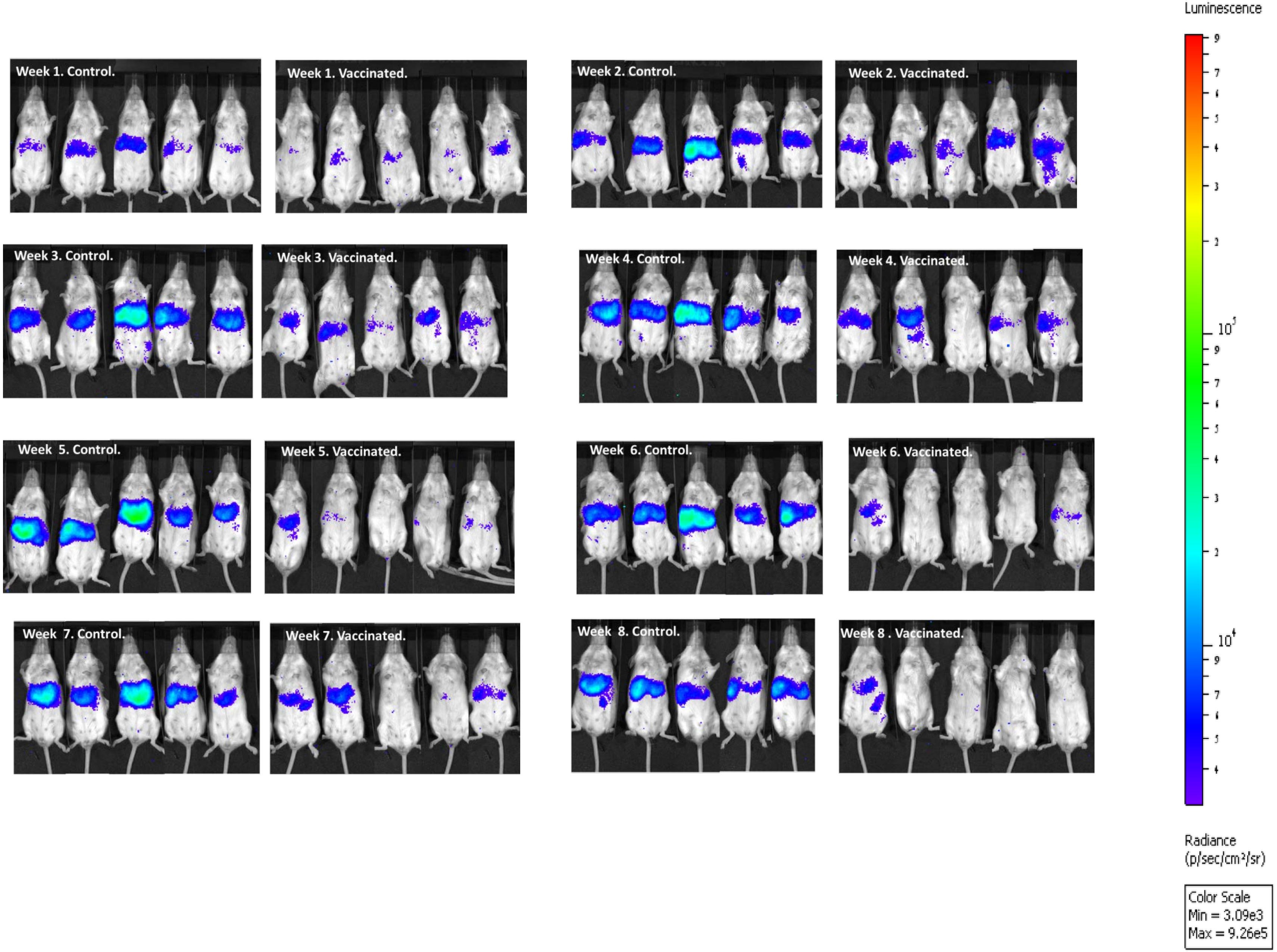
Week 1. Control.
Week 1. Vaccinated.
Week 2. Control.
Week 2. Vaccinated.
Week 3. Control.
Week 3. Vaccinated.
Week 4. Control.
Week 4. Vaccinated.
Week 5. Control.
Week 5. Vaccinated.
Week 6. Control.
Week 6. Vaccinated.
Week 7. Control.
Week 7. Vaccinated.
Week 8. Control.
Week 8 . Vaccinated.
Luminescence
Radiance (p/sec/cm²/sr)
Color Scale
Min = 3.09e3
Max = 9.26e5
